# Prescribing of glucose‐lowering medication to adults with type 2 diabetes by severe mental illness status in Scotland: A cohort study

**DOI:** 10.1111/dom.70278

**Published:** 2025-11-14

**Authors:** Jilly Adams, Shuvajit Saha, Kelly J. Fleetwood, Luke A. K. Blackbourn, Stuart J. McGurnaghan, Sarah H. Wild, Caroline A. Jackson, the Scottish Diabetes Research Network Epidemiology Group

**Affiliations:** ^1^ Usher Institute University of Edinburgh Edinburgh UK; ^2^ MRC Institute of Genetics and Cancer University of Edinburgh Edinburgh UK

**Keywords:** bipolar disorder, cohort study, depression, diabetes, glucose‐lowering medication, insulin, metformin, schizophrenia, severe mental illness

## Abstract

**Aim:**

We aimed to determine whether glucose‐lowering medication (GLM) prescribing differs by severe mental illness (SMI) status.

**Materials and methods:**

We conducted a population‐based cohort study using routinely collected linked data, including people in Scotland diagnosed with type 2 diabetes between January 2004 and March 2022. We used Cox proportional hazards models to compare the time from diabetes diagnosis to the first prescription of each of metformin and insulin in people with and without a hospital admission record of SMI (schizophrenia, bipolar disorder or depression prior to diabetes diagnosis). We adjusted for age, sex, area‐based socio‐economic deprivation, smoking status and baseline HbA1c and estimated glomerular filtration rate.

**Results:**

Among 317 761 people with type 2 diabetes, 14 600 (4.6%) had pre‐existing SMI. During a median follow‐up of 1.6 years, 10 859 (74%) of those with SMI and 219 823 (73%) without SMI were prescribed metformin. After accounting for confounders, people with SMI received their first metformin (HR 1.09, 95% CI: 1.07–1.11) and insulin (HR 1.24, 95% CI: 1.18–1.31) prescriptions at a faster rate than people without SMI. Body mass index (BMI) was missing in 21% of participants, but similar estimates were obtained when we additionally adjusted for BMI in analyses that only included people with BMI data.

**Conclusions:**

Among people with type 2 diabetes each of metformin and insulin is prescribed sooner in people with compared to those without SMI. Further research is needed to understand the reasons for this, and to investigate the implications of these differences in prescribing patterns.

## 1 INTRODUCTION

Severe mental illness (SMI), defined here as schizophrenia, bipolar disorder or major depression, is associated with a significantly higher risk of premature mortality compared with the general population.^1^, ^2^, ^3^ This is largely due to the greater burden of physical health conditions in people with SMI,^4^ particularly cardiovascular disease (CVD) and type 2 diabetes.^5^, ^6^, ^7^ People with comorbid type 2 diabetes and SMI have a higher risk of poor diabetes outcomes, but the reasons for this are not fully understood and are likely to be multifactorial.

Suboptimal receipt of routine diabetes care may contribute to the associations between SMI and diabetes outcomes. Interestingly, previous studies in the UK have found that people with SMI are generally equally, or more likely, to receive guideline‐indicated annual diabetes monitoring in primary care^8^, ^9^ and to achieve cardiovascular risk factor target levels, including for HbA1c, in the first year post‐diabetes diagnosis.^10^ A handful of studies conducted in Denmark, Germany and the USA have investigated glucose‐lowering medication (GLM) prescribing by SMI status,^11^, ^12^, ^13^, ^14^, ^15^ and have mainly focused on major depression^14^ or schizophrenia only.^12^, ^13^, ^15^ Two studies reported higher GLM initiation prescribing in people with mental illness,^11^, ^14^ whereas one study investigating initiation of second‐line GLM found no difference by schizophrenia status.^12^ Two studies reporting on the prevalence of GLM medication prescribing found increased prevalence of metformin^15^ and insulin prescribing,^13^, ^15^ although findings did not reach statistical significance in one study.^15^ These existing studies are limited by simple comparisons of proportions, small study size^12^, ^13^, ^15^ and low precision of effect estimates^13^, ^15^; use of a cross‐sectional study design^15^; and lack of control for key confounders such as duration of diabetes and socioeconomic status.^13^, ^15^

To address these shortcomings and to add novel findings from a contemporary UK setting, we investigated the initiation of prescribing of each of metformin and insulin to adults diagnosed with type 2 diabetes between 2004 and 2022 in Scotland, by SMI status.

## 2 SUBJECTS, MATERIALS AND METHODS

This article is written in accordance with REporting of Studies Conducted using Observational Routinely‐collected Data (RECORD) statement.^16^

### 2.1 Study design and data source

We conducted a retrospective cohort study using data from the Scottish Diabetes Research Network National Diabetes Study (SDRN‐NDS) dataset.^17^ This includes information on 99% of people with diagnosed diabetes in Scotland, and links information on diabetes care delivered in primary and secondary care settings, including community prescribed medication records, to other national routine health datasets, including hospital admission/day case and mortality records, via a unique individual Community Health Index number.

### 2.2 Study population

We included adults with a diagnosis of type 2 diabetes at age 30 years or over between 1 January 2004 and 28 March 2022. In order to identify people with type 2 diabetes we used the algorithmically defined diabetes type from the SDRN‐NDS dataset. The algorithm combines clinical diagnoses of diabetes (one person may have multiple, possibly inconsistent, diagnoses entered at different timepoints) with other information including the age of onset and prescription data to determine an overall record of diabetes type (see cohort description for further details^17^). We restricted the study population to those aged 30 years or over at type 2 diabetes diagnosis given the small proportion (1%) diagnosed with type 2 diabetes in Scotland aged under 30,^18^ and the risk of misclassification of diabetes type in those aged under 30.^19^ We excluded people with a prescription for metformin or insulin prior to the date of type 2 diabetes diagnosis. Prescribing data and death records were complete in SDRN‐NDS up to 30 November 2022 at the time of data extraction.

### 2.3 Severe mental illness

We ascertained pre‐existing SMI from primary or secondary diagnoses fields of acute or psychiatric hospital admission/day case records, which are available from 1981 and 1980, respectively, in Scotland. We identified people with a record of SMI prior to their date of diabetes diagnosis using International Classification of Disease (ICD) version 9 and 10 codes, with SMI defined as schizophrenia (including schizoaffective disorder; ICD‐10 F20 and F25; ICD‐9295.0–295.3, 295.6–295.9), bipolar disorder (ICD‐10 F30–F31; ICD‐9 296.0, 296.2–296.6) and major depression (ICD‐10 F32–F33; ICD‐9 296.1, 298.0, 300.4, 311). The comparison group included participants with no primary or secondary diagnosis of SMI recorded in an acute or psychiatric hospital admission record prior to diabetes diagnosis.

### 2.4 Glucose‐lowering medication prescribing

Outcomes included time from type 2 diabetes diagnosis to initial metformin prescription, and time from type 2 diabetes diagnosis to initial insulin prescription. Metformin and insulin prescriptions were determined from the inclusion of British National Formulary codes of 6.1.2.2 or 6.1.1.2 in a participant's SDRN‐NDS record at any time post‐type 2 diabetes diagnosis.

### 2.5 Covariates

Covariates at the time of diabetes diagnosis included: sex; age; year of diagnosis; smoking status (defined as current, never or ex‐smoker); area‐level deprivation, based on quintiles of the Scottish Index of Multiple Deprivation (SIMD), which uses census data on seven domains including levels of employment, income and health, to create a small‐area measure of relative deprivation; glycated haemoglobin (HbA1c, categorised as <53, 53–75 and >75 mmol/mol); and estimated glomerular filtration rate (eGFR, categorised as <30, 30–60 and ≥60 mL/min/1.73 m^2^). We used the first recorded HbA1c and eGFR measurements from the period starting from 180 days prior to the diagnosis of type 2 diabetes as the baseline HbA1c and eGFR levels for each participant.

### 2.6 Statistical methods

We summarised baseline characteristics by SMI status. Our primary analyses included participants with complete data on sex, age, smoking status, SIMD quintile, HbA1c level and eGFR level at baseline. Participants were followed up from diabetes diagnosis to the date of the first metformin or insulin prescription (as appropriate) with censoring at the earliest of the date of death (to account for the competing risk of death) or 30 November 2022 otherwise. We plotted the estimated cumulative incidence of metformin prescribing and insulin prescribing over the follow‐up period by SMI status. We obtained unadjusted and adjusted hazard ratios (HRs) for the association between SMI and each of first metformin prescribing and first insulin prescribing using Cox proportional hazards models. We obtained crude (unadjusted) estimates, with subsequent models adjusting first for age and sex, and then further adjusted for SIMD quintile, smoking status, year of type 2 diabetes diagnosis, baseline HbA1c, and eGFR. We included age and year of type 2 diabetes diagnosis in the models as mean‐centred (subtracting their respective means) continuous variables. For both continuous variables, we included linear and quadratic terms in the models based on exploratory plots that suggested this approach was appropriate for describing the relationships between these variables and the outcomes. We included other covariates, including HbA1c and eGFR, in the models as categorical variables, using standard clinical categories. We calculated generalised variance inflation factors to check for multicollinearity between the covariates. We assessed the proportional hazards assumption using log‐cumulative hazard plots and Schoenfeld residuals.

In a secondary analysis, we included the subset of participants with data on BMI at diabetes diagnosis (80% of the primary cohort), to explore how additional adjustment for BMI affected effect estimates for the association between SMI and metformin and insulin prescribing. We included BMI in the models as a mean‐centred continuous variable with both a linear and a quadratic term.

In a post hoc secondary analysis, we repeated the above steps, accounting for each SMI (schizophrenia, bipolar disorder or major depression) instead of overall SMI status. We classified people with a record of more than one SMI prior to their diabetes diagnosis by their most severe illness, with schizophrenia considered the most severe, followed by bipolar disorder and then major depression.

We conducted all analyses using R version 4.1.3.

### 2.7 Ethics approval

Approval for the linkage of the administrative health data sets and their use by approved researchers was provided by the NHS Scotland Public Benefit and Privacy Panel for Health and Social Care in Scotland (reference 1617‐0147) and ethical approval for the use of the linked database for research was obtained from the West of Scotland multi‐centre research ethics committee (reference 21/WS/0047).^20^

## 3 RESULTS

**FIGURE 1:**
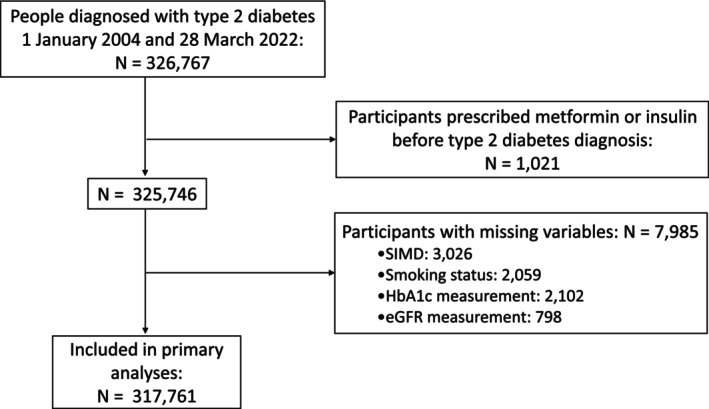
Flow diagram describing the study population, identified from the Scottish Diabetes Research Network–National Diabetes Study (SDRN‐NDS). BMI, body mass index; eGFR, estimated glomerular filtration rate; HbA1c, glycated haemoglobin; SIMD, Scottish Index of Multiple Deprivation.

Among the 325 746 people with type 2 diabetes who met the inclusion criteria (Figure 1), 15 001 (4.6%) had a history of SMI at baseline. Of these, 10 383 (3.2%) had depression, 3113 (1.0%) had schizophrenia and 1505 (0.5%) had bipolar disorder. Compared to those without SMI, participants with SMI were younger at diabetes diagnosis (mean age: 57.7 vs. 60.9 years), more likely to be female, and more commonly resided in areas of higher socioeconomic deprivation. Current smoking was almost twice as common in those with versus without SMI and mean BMI was slightly higher among participants with versus without SMI. Baseline glucose levels, summarised by median HbA1c, were 54 mmol/mol in both groups. Renal function, assessed by eGFR and HbA1c measures closest to the diagnosis of diabetes, was similar in people with and without SMI (Table 1).

**TABLE 1:** Baseline characteristics of adults diagnosed with type 2 diabetes 2004–2022 in Scotland, by comorbid severe mental illness (SMI) status.

Baseline characteristics	No SMI (*N* = 310 745)	SMI (*N* = 15 001)	Total (*N* = 325 746)
Age (years), mean (±SD)	60.9 (±12.7)	57.7 (±12.3)	60.8 (±12.7)
Female, *n* (%)	133 640 (43.0)	8315 (55.4)	141 955 (43.6)
SIMD quintile, *n* (%)			
1 (most deprived)	74 299 (23.9)	5163 (34.4)	79 462 (24.4)
2	71 002 (22.8)	3755 (25.0)	74 757 (22.9)
3	63 597 (20.5)	2845 (19.0)	66 442 (20.4)
4	55 592 (17.9)	1884 (12.6)	57 476 (17.6)
5 (least deprived)	43 360 (14.0)	1223 (8.2)	44 583 (13.7)
Missing	2895 (0.9)	131 (0.9)	3026 (0.9)
Smoking status, *n* (%)			
Current smoker	61 713 (19.9)	5931 (39.5)	67 644 (20.8)
Ex‐smoker	106 808 (34.4)	4250 (28.3)	111 058 (34.1)
Never smoked	140 244 (45.1)	4718 (31.5)	144 962 (44.5)
Missing	1980 (0.64)	102 (0.68)	2082 (0.64)
BMI (kg/m^2^), mean (SD)	33.0 (6.9)	34.6 (7.5)	33 (6.9)
BMI category (kg/m^2^), *n* (%)			
<25	21 745 (7.0)	857 (5.7)	22 602 (6.9)
25–29	68 395 (22.0)	2421 (16.1)	70 816 (21.7)
30–34	76 858 (24.7)	3542 (23.6)	80 400 (24.7)
35–39	44 878 (14.4)	2587 (17.2)	47 465 (14.6)
40+	35 287 (11.4)	2411 (16.1)	37 698 (11.6)
Missing	63 582 (20.5)	3183 (21.2)	66 765 (20.5)
HbA1c (mmol/mol), median (IQR)	54.0 (48.0, 74.0)	54.0 (48.0, 72.0)	54.0 (48.0, 74.0)
HbA1c (%; mmol/mol)			
<7.0; <53	135 403 (43.6)	6833 (45.6)	142 236 (43.7)
7.0–9.0; 53–75	100 218 (32.3)	4777 (31.8)	104 995 (32.2)
>9.0; >75	72 681 (23.4)	3261 (21.7)	75 942 (23.3)
Missing	2443 (0.8)	130 (0.9)	2573 (0.8)
eGFR (mL/min/1.73 m^2^), median (IQR)	82.7 (67.4, 95.8)	85.3 (69.4, 98.4)	82.8 (67.5, 96.0)
eGFR (mL/min/1.73 m^2^), *n* (%)			
<30	2767 (0.9)	148 (1.0)	2915 (0.9)
30–60	45 549 (14.7)	1954 (13.0)	47 503 (14.6)
≥60	260 466 (83.8)	12 786 (85.2)	273 252 (83.9)
Missing	1963 (0.6)	113 (0.8)	2076 (0.6)

Abbreviations: BMI, body mass index; eGFR, estimated glomerular filtration rate; HbA1c, glycated haemoglobin; IQR, interquartile range; SD, standard deviation; SIMD, Scottish index of multiple deprivation.

**FIGURE 2:**
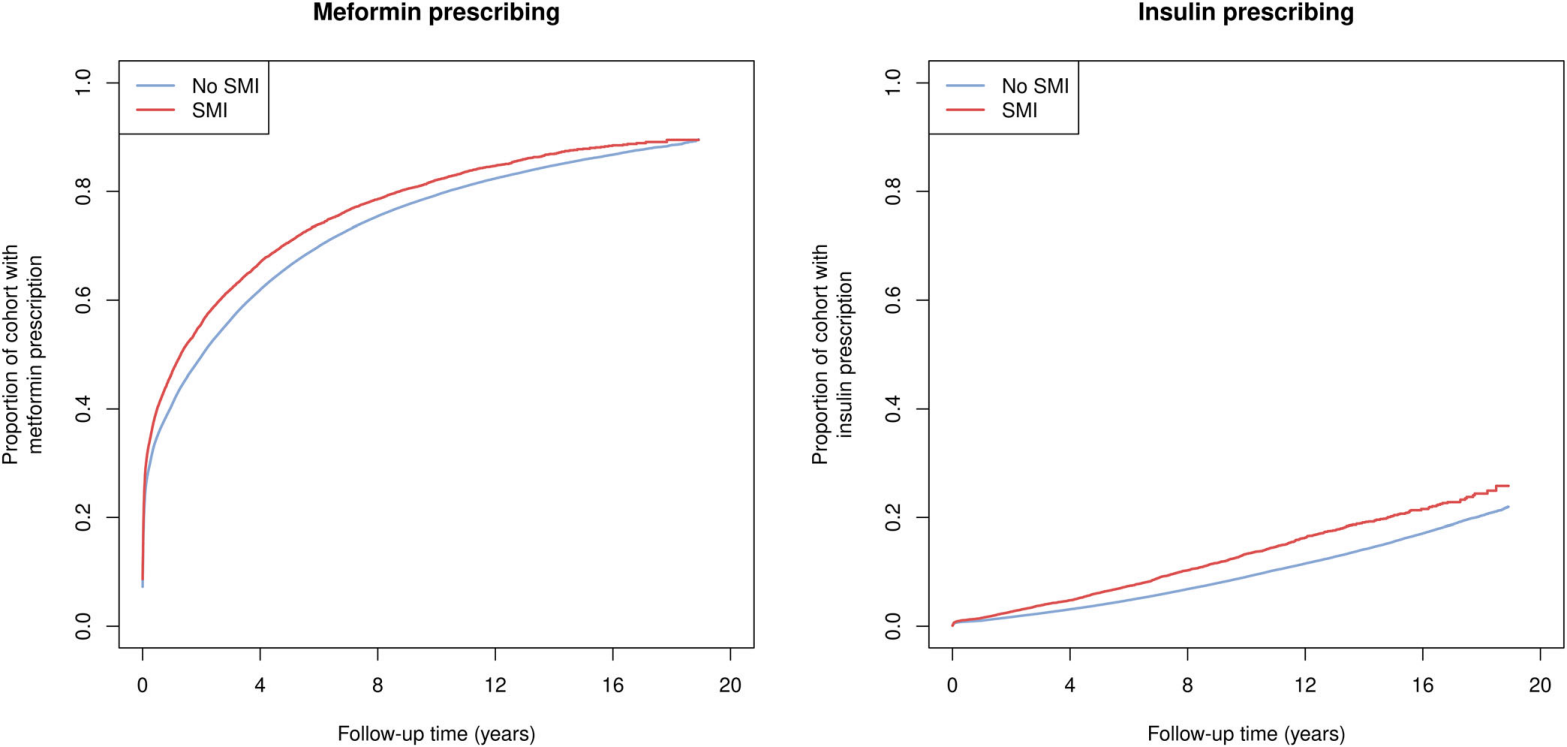
Estimated cumulative incidence of metformin prescribing and insulin prescribing during follow‐up, by severe mental illness (SMI) status.

After excluding participants with missing data, 317 761 people (97.6%) were included in the primary analyses (Figure 1), of whom 14 600 had an SMI. For metformin, median follow‐up was 1.6 years, with 230 682 (72.6%) participants prescribed metformin before the end of follow‐up, 31 099 (9.8%) dying prior to receiving a metformin prescription and 55 980 (17.6%) censored at the end of follow‐up. Likewise for insulin, median follow‐up was 7.6 years, with 26 537 (8.4%) participants prescribed insulin before the end of follow‐up, 70 947 (22.3%) dying prior to receiving an insulin prescription and 220 277 (69.3%) censored at the end of follow‐up. Cumulative incidence curves illustrate that, following a type 2 diabetes diagnosis, participants with SMI were prescribed both metformin and insulin sooner after diagnosis of diabetes than those without SMI (Figure 2). At 2 years post‐diabetes diagnosis, 49.7% of people without SMI had received a metformin prescription, compared to 55.7% of those with SMI. At 10 years, 9.1% of people without SMI had received an insulin prescription, versus 13.3% of those with SMI.

**FIGURE 3:**
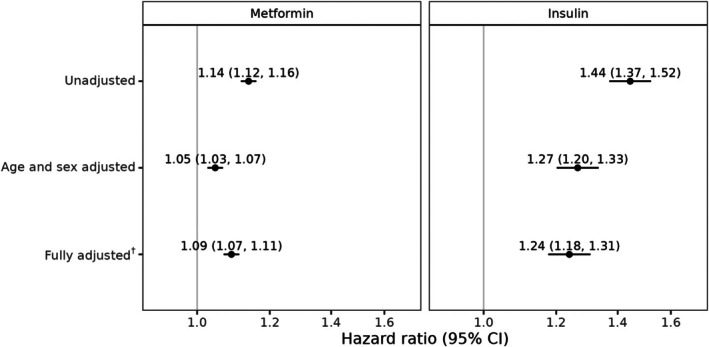
Unadjusted and adjusted hazard ratios with 95% confidence intervals for the association between severe mental illness and receipt of each of metformin and insulin among people diagnosed with diabetes 2004–2022, Scotland. (†) Fully adjusted model includes age, sex, area‐based deprivation, smoking status, year of type 2 diabetes diagnosis, baseline HbA1c, and estimated glomerular filtration rate.

In the unadjusted model, the rate of initiation of metformin prescribing was 14% higher (HR 1.14, 95% CI: 1.12–1.16) in people with versus without SMI, while the rate of initiation of insulin was 44% higher (HR 1.44, 95% CI: 1.37–1.52) in those with versus without SMI. After adjusting for age and sex, effect estimates attenuated, but SMI remained associated with higher rates of initiation of metformin and insulin. Upon further adjustment for area‐level deprivation, smoking status, year of type 2 diabetes diagnosis and baseline HbA1c and eGFR, the rates of metformin and insulin initiation were 9% (HR 1.09, 95% CI: 1.07–1.11) and 24% higher (HR 1.24, 95% CI: 1.18–1.31), respectively, in people with versus without SMI (Figure 3). There was no substantial multicollinearity between the covariates.

In the serially adjusted multivariate models, adjusting for age had the greatest impact on the association between SMI and time to first prescription of both metformin and insulin, as younger age was associated with shorter time to metformin and insulin prescribing and people with SMI were younger than those without SMI. Adjusting for baseline HbA1c also had a relatively large impact on the hazard ratios, especially for time to first prescription of metformin. Baseline HbA1c was slightly lower in people with SMI and higher baseline HbA1c was associated with shorter time to first prescription of each medication (Tables S1 and S2, Supporting Information).

In secondary analyses, where we additionally adjusted models for BMI among those in whom BMI data was available, effect estimates were almost identical to those obtained in the primary analysis, indicating that additional adjustment for BMI had minimal influence on the observed associations (Tables S3 and S4). Again, there was no substantial multicollinearity between the covariates.

For the post hoc secondary analysis where we examined schizophrenia, bipolar disorder and major depression separately the baseline characteristics are provided in Table S5. Based on the model adjusted for age, sex, area‐level deprivation, smoking status, year of type 2 diabetes diagnosis and baseline HbA1c and eGFR, the rate of metformin initiation was 7% higher (HR 1.07, 95% CI: 1.01–1.14) in people with bipolar disorder and 13% higher (HR 1.13, 95% CI: 1.10–1.15) in people with major depression, compared to those without SMI. Based on the same model, there was no evidence of a difference in metformin initiation rates between people with schizophrenia and those without SMI (HR 0.99, 95% CI: 0.95–1.03) (Table S6). For insulin, based on the model adjusted for age, sex, area‐level deprivation, smoking status, year of type 2 diabetes diagnosis and baseline HbA1c and eGFR, the rate of initiation was 22% higher (HR 1.22, 95% CI: 1.04–1.44) in people with bipolar disorder, 43% higher in people with major depression (HR 1.43, 95% CI: 1.35–1.52) but 22% lower (HR 0.78, 95% CI: 0.69–0.89) in people with schizophrenia (Table S7).

## 4 DISCUSSION

Among adults with type 2 diabetes in Scotland, each of metformin and insulin prescribing is initiated earlier among those with versus without SMI. These findings remained statistically significant after adjusting for sex, age, smoking status, SIMD quintile, baseline HbA1c level, baseline eGFR level and year of diagnosis. Further adjustment for BMI made little difference to these results in the sub‐cohort in whom BMI at baseline was recorded.

Our results align with those of Bakkedal et al., the most comparable to our study in terms of study design, exposure and outcome measures. They also found that, during a maximum 10‐year follow‐up of 42 854 adults with type 2 diabetes in Denmark, people with SMI (defined as schizophrenia, bipolar disorder, unipolar depression, other affective disorders or personality disorders) were more likely to be prescribed each of metformin and insulin than those without SMI.^11^ Similarly, another Danish study that investigated GLM prescribing by comorbid major depression status during the first year post‐diabetes diagnosis found that major depression was associated with earlier initiation of any GLM (the majority of which was metformin).^14^ The magnitude of association was slightly smaller than in our study, perhaps due to methodological differences, including mental health condition examined, analysis of composite GLM, and confounders adjusted for. Insulin prescribing was not reported, possibly due to the short follow‐up period.^14^ Our post hoc secondary analyses suggest that associations with metformin and insulin prescribing may differ by SMI disorder, with findings differing for schizophrenia as compared to bipolar disorder and major depression. These findings add further novel insight to the most robust existing evidence, but require further investigation in future studies. Our study is less comparable to a third Danish matched cohort study^12^ that focused on initiation of second‐line GLM among people already prescribed metformin, and a German case–control study^13^ which examined prescribing patterns among those currently prescribed at least one GLM. Both of these studies compared second or subsequent line prescribing in people with versus without schizophrenia. The former reported no difference in second‐line GLM prescribing, including insulin,^12^ while the latter found a higher prevalence of insulin prescribing in people with versus without schizophrenia.^13^ While also limited by small sample size and cross‐sectional design, Weiss found a higher prevalence of any GLM and of insulin specifically, although effect estimates did not reach statistical significance possibly due to low study power.^15^

Earlier initiation of metformin prescribing for adults with SMI and type 2 diabetes could be regarded as beneficial. In light of the barriers many adults with SMI face to engaging in behaviours which could help blood glucose levels,^21^, ^22^, ^23^ prescribing metformin to these individuals more quickly after a type 2 diabetes diagnosis could assist with improving glucose levels. However, it may also reflect reduced participation in or healthcare provider‐related optimism about successful lifestyle intervention. Given that metformin may mitigate the weight‐inducing side effects of some psychotropic medications, the threshold for initiation of metformin may be lower in people with SMI. Earlier metformin initiation might help explain findings from a previous Scottish study and other research which indicate people with SMI are equally, or more, likely to achieve HbA1c target levels following diabetes diagnosis.^10^, ^14^, ^24^, ^25^ The reasons for earlier insulin initiation in adults with SMI are not yet clear. Evidence of earlier insulin initiation in people with SMI may seem surprising given reports of similar or better glucose levels in people with SMI. However, longer term patterns of glucose levels by SMI status are not well described. One UK study that examined repeated HbA1c values over time found that, although average HbA1c levels did not differ in people with versus without SMI, a greater proportion of patients with SMI experienced extreme high or low values that may affect prescribing decisions.^26^

Our study has a number of strengths. The study population is representative of the Scottish population with diagnosed diabetes, since the dataset includes approximately 99% of adults with a diabetes diagnosis who lived in Scotland between 1 January 2004 and 28 March 2022. To our knowledge it is by far the largest study to date to examine metformin and insulin prescribing by SMI status, overcoming shortcomings of size and precision that limit most previous studies. It also includes the longest follow‐up period to date among cohort studies that have investigated prescribing of GLM to adults by SMI status. Furthermore, we objectively ascertained both exposures and outcomes, and adjusted for a range of key confounding factors in our analyses.

Our study does have some limitations. We ascertained SMI status from hospital admission records, potentially misclassifying individuals with SMI who had never been admitted to hospital as not having SMI and we did not account for receipt of an SMI diagnosis subsequent to diabetes diagnosis. Resulting misclassification of SMI status may have diluted associations between SMI status and time to metformin and insulin prescribing. In addition, ascertaining SMI status from hospital records also means that we have only identified adults with particularly severe SMI. Our findings may therefore not be generalisable to all adults with SMI. While we adjusted for a number of key confounding factors, there may still be residual confounding, including from incomplete adjustment of smoking behaviour, with heavy smoking very common in people with SMI.^27^ We also did not account for changes over time in HbA1c and eGFR, which were beyond the scope of the present study. It is important to additionally note that the nature of the study means we cannot draw causal conclusions relating to SMI and prescribing of GLM. Finally, it was beyond the scope and resources of the present study to expand our analyses to examine prescribing of glucose‐lowering medication other than metformin and insulin.

Our findings make an important contribution to the paucity of studies on SMI and GLM prescribing among people with type 2 diabetes and have implications for future research and clinical practice. Further studies, both quantitative and qualitative, should seek to identify reasons for earlier metformin and insulin prescribing to adults with versus without SMI and opportunities for appropriate postponing of insulin initiation in those with SMI. Additional research, using well‐powered studies is also needed to further investigate how GLM prescribing differs by SMI disorder, and whether GLM prescribing is influenced by psychotropic medication prescribing. Another key step is to extend the present research to investigate the role of glucose lowering treatment in the risk of diabetes complications in people with and without SMI in Scotland. This would also inform whether GLM prescribing patterns partly explain the greater risk of severe hypoglycaemia and hyperglycaemia associated with SMI observed in other studies.^28^, ^29^ It would also be beneficial to explore whether the prescribing of GLM other than metformin and insulin varies by SMI status, particularly in response to recent changes to clinical guidance on GLM prescribing for people with type 2 diabetes.^30^ Recent analyses of Scottish data indicate that individuals with type 2 diabetes in Scotland now tend to be prescribed newer antidiabetic drugs as a second‐line treatment or as part of a combination first‐line treatment with metformin.^31^ There is also scope to examine the effectiveness of GLM prescribing optimisation tools^32^ for particular subgroups, including people with SMI. Meanwhile, improved integrated mental‐physical health care for people with SMI is urgently needed to ensure that health care providers, including primary care physicians and community mental health teams effectively support people with SMI diagnosed with diabetes to achieve optimal self‐management, to minimise transition to insulin prescribing and reduce both acute and chronic diabetes complications.

## 5 CONCLUSION

In conclusion, we found that adults with diabetes and pre‐existing SMI in Scotland are prescribed each of metformin and insulin sooner after the diagnosis of diabetes than adults with type 2 diabetes only. Explanations, for and implications of, these prescribing patterns should be investigated in future studies.

## AUTHOR CONTRIBUTIONS

CJ conceived the study; JA, CJ, SW and KF designed the study; JA, SS and KF conducted the analyses; LB and SM conducted data acquisition, cleaning and processing; all authors contributed to the interpretation of findings; JA, SS and CJ wrote the draft manuscript and all authors commented on the final draft.

## FUNDING INFORMATION

Funding for the Scottish Diabetes Research Network is provided by the National Health Service (NHS) Research Scotland, a collaborative effort involving Scottish NHS Boards and the Chief Scientist Office of the Scottish Government. KF, SW and CJ are supported by the UKRI‐funded University of Edinburgh Hub for Metabolic Psychiatry (Ref MR/Z503563/1), within the UK Mental Health Platform (Ref MR/Z000548/1).

## CONFLICT OF INTEREST STATEMENT

The authors declare no conflicts of interest.

## Supporting information


**Table S1.** Results of unadjusted and serial adjustments to the multivariate model used to evaluate the association between SMI and time to first metformin prescription in the primary cohort (*n* = 317 761).
**Table S2.** Results of unadjusted and serial adjustments to the multivariate model used to evaluate the association between SMI and time to first insulin prescription in the primary cohort (*n* = 317 761).
**Table S3.** Results of unadjusted and serial adjustments to the multivariate model used to evaluate the association between SMI and time to first metformin prescription in the secondary cohort where BMI was additionally adjusted for (*N* = 254 893).
**Table S4.** Results of unadjusted and serial adjustments to the multivariate model used to evaluate the association between SMI and time to first insulin prescription in the secondary cohort, where BMI was additionally adjusted for (*N* = 254 893).
**Table S5.** Baseline characteristics of adults diagnosed with type 2 diabetes 2004–2022 in Scotland, by each severe mental illness (SMI).
**Table S6.** Results of unadjusted and serial adjustments to the multivariate model used to evaluate the association between each SMI and time to first metformin prescription in the primary cohort (*n* = 317 761).
**Table S7.** Results of unadjusted and serial adjustments to the multivariate model used to evaluate the association between each SMI and time to first insulin prescription in the primary cohort (*n* = 317 761).

## Data Availability

Data are available via application to the Scottish Diabetes Research Network Epidemiology Group.
