# Supplementary material for: Prescribing of glucose‐lowering medication to adults with type 2 diabetes by severe mental illness status in Scotland: A cohort study
Source: Diabetes Obes Metab. 2025 Nov 14;28(2):1049–56. doi: 10.1111/dom.70278 (PMC12803641; doi:10.1111/dom.70278)
Supplement: Supplementary file 1 — Table S1. Results of unadjusted and serial adjustments to the multivariate model used to evaluate the association between SMI and time to first metformin prescription in the primary cohort (n = 317 761). Table S2. Results of unadjusted and serial adjustments to the multivariate model used to evaluate the association between SMI and time to first insulin prescription in the primary cohort (n = 317 761). Table S3. Results of unadjusted and serial adjustments to the multivariate model used to evaluate the association between SMI and time to first metformin prescription in the secondary cohort where BMI was additionally adjusted for (N = 254 893). Table S4. Results of unadjusted and serial adjustments to the multivariate model used to evaluate the association between SMI and time to first insulin prescription in the secondary cohort, where BMI was additionally adjusted for (N = 254 893). Table S5. Baseline characteristics of adults diagnosed with type 2 diabetes 2004–2022 in Scotland, by each severe mental illness (SMI). Table S6. Results of unadjusted and serial adjustments to the multivariate model used to evaluate the association between each SMI and time to first metformin prescription in the primary cohort (n = 317 761). Table S7. Results of unadjusted and serial adjustments to the multivariate model used to evaluate the association between each SMI and time to first insulin prescription in the primary cohort (n = 317 761). [file DOM-28-1049-s001.docx]

**Supplementary material**

**Prescribing of glucose-lowering medication to adults with type 2 diabetes by severe mental illness status in Scotland: a retrospective cohort study**

Jilly Adams^1,3^, Shuvajit Saha^1,3^, Kelly J Fleetwood^1,3^, Luke A K Blackbourn^2,3^, Stuart J. McGurnaghan^2,3^, Sarah H Wild^1,3^, Caroline A Jackson^1,3*^

^1^Usher Institute, University of Edinburgh, Edinburgh, UK

^2^MRC Institute of Genetics and Cancer, University of Edinburgh, Edinburgh, UK.

^3^On behalf of the Scottish Diabetes Research Network Epidemiology Group

Contents

[Supplementary Table S1: Results of unadjusted and serial adjustments to the multivariate model used to evaluate the association between SMI and time to first metformin prescription in the primary cohort (n=317,761) 3](#_Toc211868389)

[Supplementary Table S2: Results of unadjusted and serial adjustments to the multivariate model used to evaluate the association between SMI and time to first insulin prescription in the primary cohort (n=317,761) 3](#_Toc211868390)

[Supplementary Table S3: Results of unadjusted and serial adjustments to the multivariate model used to evaluate the association between SMI and time to first metformin prescription in the secondary cohort where BMI was additionally adjusted for (N=254,893) 4](#_Toc211868391)

[Supplementary Table S4: Results of unadjusted and serial adjustments to the multivariate model used to evaluate the association between SMI and time to first insulin prescription in the secondary cohort, where BMI was additionally adjusted for (N=254,893) 4](#_Toc211868392)

[Supplementary Table S5: Baseline characteristics of adults diagnosed with type 2 diabetes 2004-2022 in Scotland, by each severe mental illness (SMI) 5](#_Toc211868393)

[Supplementary Table S6: Results of unadjusted and serial adjustments to the multivariate model used to evaluate the association between each SMI and time to first metformin prescription in the primary cohort (n=317,761) 6](#_Toc211868394)

[Supplementary Table S7: Results of unadjusted and serial adjustments to the multivariate model used to evaluate the association between each SMI and time to first insulin prescription in the primary cohort (n=317,761) 6](#_Toc211868395)

# Supplementary Table S1: Results of unadjusted and serial adjustments to the multivariate model used to evaluate the association between SMI and time to first metformin prescription in the primary cohort (n=317,761)

| **Models** | **Hazard ratio** | **95% CI (lower)** | **95% CI (upper)** |
| --- | --- | --- | --- |
| Model 1 (Unadjusted) | 1.14 | 1.12 | 1.16 |
| Model 2^a^ | 1.04 | 1.02 | 1.06 |
| Model 3^b^ | 1.05 | 1.03 | 1.07 |
| Model 4^c^ | 1.02 | 1.00 | 1.04 |
| Model 5^d^ | 1.02 | 1.00 | 1.04 |
| Model 6^e^ | 1.01 | 0.99 | 1.03 |
| Model 7^f^ | 1.08 | 1.06 | 1.10 |
| Model 8^g^ | 1.09 | 1.07 | 1.11 |

^a^ Adjusted for age

^b^ Adjusted for age and sex

^c^ Adjusted for age, sex and SIMD quantile

^d^ Adjusted for age, sex, SIMD quantile and smoking

^e^ Adjusted for age, sex, SIMD quantile, smoking and year of type 2 diabetes diagnosis

^f^ Adjusted for age, sex, SIMD quantile, smoking, year of type 2 diabetes diagnosis and baseline HbA1c

^g^ Adjusted for age, sex, SIMD quantile, smoking, year of type 2 diabetes diagnosis, baseline HbA1c and baseline eGFR

# Supplementary Table S2: Results of unadjusted and serial adjustments to the multivariate model used to evaluate the association between SMI and time to first insulin prescription in the primary cohort (n=317,761)

| **Models** | **Hazard ratio** | **95% CI (lower)** | **95% CI (upper)** |
| --- | --- | --- | --- |
| Model 1 (Unadjusted) | 1.44 | 1.37 | 1.52 |
| Model 2^a^ | 1.30 | 1.24 | 1.37 |
| Model 3^b^ | 1.27 | 1.20 | 1.33 |
| Model 4^c^ | 1.25 | 1.19 | 1.32 |
| Model 5^d^ | 1.19 | 1.13 | 1.25 |
| Model 6^e^ | 1.20 | 1.14 | 1.26 |
| Model 7^f^ | 1.26 | 1.19 | 1.33 |
| Model 8^g^ | 1.24 | 1.18 | 1.31 |

^a^ Adjusted for age

^b^ Adjusted for age and sex

^c^ Adjusted for age, sex and SIMD quantile

^d^ Adjusted for age, sex, SIMD quantile and smoking

^e^ Adjusted for age, sex, SIMD quantile, smoking and year of type 2 diabetes diagnosis

^f^ Adjusted for age, sex, SIMD quantile, smoking, year of type 2 diabetes diagnosis and baseline HbA1c

^g^ Adjusted for age, sex, SIMD quantile, smoking, year of type 2 diabetes diagnosis, baseline HbA1c and baseline eGFR

# Supplementary Table S3: Results of unadjusted and serial adjustments to the multivariate model used to evaluate the association between SMI and time to first metformin prescription in the secondary cohort where BMI was additionally adjusted for (N=254,893)

| **Models** | **Hazard ratio** | **95% CI (lower)** | **95% CI (upper)** |
| --- | --- | --- | --- |
| Model 1 (Unadjusted) | 1.11 | 1.09 | 1.13 |
| Model 2^a^ | 0.99 | 0.97 | 1.01 |
| Model 3^b^ | 1.00 | 0.97 | 1.02 |
| Model 4^c^ | 0.97 | 0.95 | 1.00 |
| Model 5^d^ | 0.97 | 0.95 | 0.99 |
| Model 6^e^ | 0.97 | 0.95 | 0.99 |
| Model 7^f^ | 1.05 | 1.03 | 1.07 |
| Model 8^g^ | 1.06 | 1.03 | 1.08 |
| Model 9^h^ | 1.05 | 1.03 | 1.07 |

^a^ Adjusted for age

^b^ Adjusted for age and sex

^c^ Adjusted for age, sex and SIMD quantile

^d^ Adjusted for age, sex, SIMD quantile and smoking

^e^ Adjusted for age, sex, SIMD quantile, smoking and year of type 2 diabetes diagnosis

^f^ Adjusted for age, sex, SIMD quantile, smoking, year of type 2 diabetes diagnosis and baseline HbA1c

^g^ Adjusted for age, sex, SIMD quantile, smoking, year of type 2 diabetes diagnosis, baseline HbA1c and baseline eGFR

^h^ Adjusted for age, sex, SIMD quantile, smoking, year of type 2 diabetes diagnosis, baseline HbA1c, baseline eGFR and BMI

# Supplementary Table S4: Results of unadjusted and serial adjustments to the multivariate model used to evaluate the association between SMI and time to first insulin prescription in the secondary cohort, where BMI was additionally adjusted for (N=254,893)

| **Models** | **Hazard ratio** | **95% CI (lower)** | **95% CI (upper)** |
| --- | --- | --- | --- |
| Model 1 (Unadjusted) | 1.44 | 1.35 | 1.52 |
| Model 2^a^ | 1.28 | 1.21 | 1.36 |
| Model 3^b^ | 1.25 | 1.18 | 1.32 |
| Model 4^c^ | 1.24 | 1.16 | 1.31 |
| Model 5^d^ | 1.17 | 1.10 | 1.24 |
| Model 6^e^ | 1.19 | 1.12 | 1.26 |
| Model 7^f^ | 1.26 | 1.18 | 1.33 |
| Model 8^g^ | 1.24 | 1.17 | 1.31 |
| Model 9^h^ | 1.26 | 1.19 | 1.34 |

^a^ Adjusted for age

^b^ Adjusted for age and sex

^c^ Adjusted for age, sex and SIMD quantile

^d^ Adjusted for age, sex, SIMD quantile and smoking

^e^ Adjusted for age, sex, SIMD quantile, smoking and year of type 2 diabetes diagnosis

^f^ Adjusted for age, sex, SIMD quantile, smoking, year of type 2 diabetes diagnosis and baseline HbA1c

^g^ Adjusted for age, sex, SIMD quantile, smoking, year of type 2 diabetes diagnosis, baseline HbA1c and baseline eGFR

^h^ Adjusted for age, sex, SIMD quantile, smoking, year of type 2 diabetes diagnosis, baseline HbA1c, baseline eGFR and BMI

# Supplementary Table S5: Baseline characteristics of adults diagnosed with type 2 diabetes 2004-2022 in Scotland, by each severe mental illness (SMI)

| **Baseline Characteristics** | **No SMI (N=310,745)** | **Schizophrenia (N=3113)** | **Bipolar disorder (N=1505)** | **Depression (N=10,383)** |
| --- | --- | --- | --- | --- |
| Age (years), mean (SD) | 60.9 (12.7) | 53.1 (11.9) | 57.7 (11.9) | 59.1 (12.1) |
| Female, n (%) | 133640 (43.0%) | 1231 (39.5%) | 899 (59.7%) | 6185 (59.6%) |
| SIMD quintile, n (%) |  |  |  |  |
| 1 (most deprived) | 74299 (23.9%) | 1168 (37.5%) | 463 (30.8%) | 3532 (34.0%) |
| 2 | 71002 (22.8%) | 798 (25.6%) | 339 (22.5%) | 2618 (25.2%) |
| 3 | 63597 (20.5%) | 569 (18.3%) | 297 (19.7%) | 1979 (19.1%) |
| 4 | 55592 (17.9%) | 352 (11.3%) | 243 (16.1%) | 1289 (12.4%) |
| 5 (least deprived) | 43360 (14.0%) | 202 (6.5%) | 152 (10.1%) | 869 (8.4%) |
| *Missing* | *2895 (0.9%)* | *24 (0.8%)* | *11 (0.7%)* | *96 (0.9%)* |
| Smoking status, n (%) |  |  |  |  |
| Current smoker | 61713 (19.9%) | 1599 (51.4%) | 571 (37.9%) | 3761 (36.2%) |
| Ex-smoker | 106808 (34.4%) | 678 (21.8%) | 448 (29.8%) | 3124 (30.1%) |
| Never smoked | 140244 (45.1%) | 803 (25.8%) | 475 (31.6%) | 3440 (33.1%) |
| *Missing* | *1980 (0.6%)* | *33 (1.1%)* | *11 (0.7%)* | *58 (0.6%)* |
| BMI (kg/m2), mean (SD) | 33.0 (6.9) | 34.2 (7.0) | 34.6 (7.1) | 34.7 (7.8) |
| BMI category (kg/m2), n (%) |  |  |  |  |
| <25 | 21745 (7.0%) | 189 (6.1%) | 78 (5.2%) | 590 (5.7%) |
| 25-29 | 68395 (22.0%) | 493 (15.8%) | 240 (15.9%) | 1688 (16.3%) |
| 30-34 | 76858 (24.7%) | 791 (25.4%) | 369 (24.5%) | 2382 (22.9%) |
| 35-39 | 44878 (14.4%) | 555 (17.8%) | 262 (17.4%) | 1770 (17.0%) |
| 40+ | 35287 (11.4%) | 466 (15.0%) | 242 (16.1%) | 1703 (16.4%) |
| *Missing* | *63582 (20.5%)* | *619 (19.9%)* | *314 (20.9%)* | *2250 (21.7%)* |
| HbA1c (mmol/mol),  median (IQR) | 54.0 [48.0, 74.0] | 54.8 [46.2, 77.0] | 53.0 [46.0, 68.0] | 54.0 [48.0, 70.0] |
| HbA1c (%; mmol/mol) |  |  |  |  |
| <7.0; <53 | 135403 (43.6%) | 1380 (44.3%) | 718 (47.7%) | 4735 (45.6%) |
| 7.0-9.0; 53-75 | 100218 (32.3%) | 894 (28.7%) | 482 (32.0%) | 3401 (32.8%) |
| >9.0; >75 | 72681 (23.4%) | 814 (26.1%) | 292 (19.4%) | 2155 (20.8%) |
| *Missing* | *2443 (0.8%)* | *25 (0.8%)* | *13 (0.9%)* | *92 (0.9%)* |
| eGFR (ml/min/1.73m2),  median (IQR) | 82.7 [67.4, 95.8] | 89.8 [73.8, 102.5] | 83.0 [66.2, 97.0] | 84.5 [68.8, 97.4] |
| eGFR (ml/min/1.73m2), n (%) |  |  |  |  |
| <30 | 2767 (0.9%) | 23 (0.7%) | 24 (1.6%) | 101 (1.0%) |
| 30-60 | 45549 (14.7%) | 315 (10.1%) | 228 (15.1%) | 1411 (13.6%) |
| ≥60 | 260466 (83.8%) | 2741 (88.1%) | 1245 (82.7%) | 8800 (84.8%) |
| *Missing* | *1963 (0.6%)* | *34 (1.1%)* | *8 (0.5%)* | *71 (0.7%)* |

BMI = body mass index; eGFR = estimated glomerular filtration rate; HbA1c = glycated haemoglobin; IQR = interquartile range; SD = standard deviation; SIMD = Scottish index of multiple deprivation

# Supplementary Table S6: Results of unadjusted and serial adjustments to the multivariate model used to evaluate the association between each SMI and time to first metformin prescription in the primary cohort (n=317,761)

|  | **Hazard ratio (95% CI)** | | |
| --- | --- | --- | --- |
| **Models** | **Schizophrenia** | **Bipolar disorder** | **Depression** |
| Model 1 (Unadjusted) | 1.14 (1.09, 1.19) | 1.07 (1.01, 1.13) | 1.15 (1.12, 1.17) |
| Model 2^a^ | 0.94 (0.90, 0.98) | 0.98 (0.93, 1.04) | 1.08 (1.06, 1.11) |
| Model 3^b^ | 0.94 (0.90, 0.98) | 0.99 (0.93, 1.05) | 1.09 (1.07, 1.12) |
| Model 4^c^ | 0.91 (0.88, 0.95) | 0.98 (0.92, 1.04) | 1.07 (1.04, 1.09) |
| Model 5^d^ | 0.91 (0.87, 0.95) | 0.98 (0.92, 1.04) | 1.07 (1.04, 1.09) |
| Model 6^e^ | 0.91 (0.87, 0.95) | 0.97 (0.91, 1.03) | 1.05 (1.02, 1.07) |
| Model 7^f^ | 0.98 (0.94, 1.03) | 1.05 (0.99, 1.12) | 1.12 (1.10, 1.15) |
| Model 8^g^ | 0.99 (0.95, 1.03) | 1.07 (1.01, 1.14) | 1.13 (1.10, 1.15) |

^a^ Adjusted for age

^b^ Adjusted for age and sex

^c^ Adjusted for age, sex and SIMD quantile

^d^ Adjusted for age, sex, SIMD quantile and smoking

^e^ Adjusted for age, sex, SIMD quantile, smoking and year of type 2 diabetes diagnosis

^f^ Adjusted for age, sex, SIMD quantile, smoking, year of type 2 diabetes diagnosis and baseline HbA1c

^g^ Adjusted for age, sex, SIMD quantile, smoking, year of type 2 diabetes diagnosis, baseline HbA1c and baseline eGFR

# Supplementary Table S7: Results of unadjusted and serial adjustments to the multivariate model used to evaluate the association between each SMI and time to first insulin prescription in the primary cohort (n=317,761)

|  | **Hazard ratio (95% CI)** | | |
| --- | --- | --- | --- |
| **Models** | **Schizophrenia** | **Bipolar disorder** | **Depression** |
| Model 1 (Unadjusted) | 1.06 (0.93, 1.20) | 1.39 (1.18, 1.64) | 1.59 (1.49, 1.68) |
| Model 2^a^ | 0.81 (0.72, 0.92) | 1.29 (1.10, 1.51) | 1.51 (1.42, 1.60) |
| Model 3^b^ | 0.82 (0.72, 0.93) | 1.24 (1.05, 1.46) | 1.45 (1.36, 1.54) |
| Model 4^c^ | 0.81 (0.71, 0.91) | 1.23 (1.05, 1.45) | 1.43 (1.35, 1.52) |
| Model 5^d^ | 0.75 (0.66, 0.85) | 1.17 (1.00, 1.38) | 1.37 (1.29, 1.46) |
| Model 6^e^ | 0.76 (0.67, 0.86) | 1.18 (1.00, 1.38) | 1.38 (1.30, 1.47) |
| Model 7^f^ | 0.79 (0.69, 0.89) | 1.27 (1.08, 1.49) | 1.45 (1.37, 1.54) |
| Model 8^g^ | 0.78 (0.69, 0.89) | 1.22 (1.04, 1.44) | 1.43 (1.35, 1.52) |

^a^ Adjusted for age

^b^ Adjusted for age and sex

^c^ Adjusted for age, sex and SIMD quantile

^d^ Adjusted for age, sex, SIMD quantile and smoking

^e^ Adjusted for age, sex, SIMD quantile, smoking and year of type 2 diabetes diagnosis

^f^ Adjusted for age, sex, SIMD quantile, smoking, year of type 2 diabetes diagnosis and baseline HbA1c

^g^ Adjusted for age, sex, SIMD quantile, smoking, year of type 2 diabetes diagnosis, baseline HbA1c and baseline eGFR
